# Medium-Chain Acyl-CoA Dehydrogenase Deficiency in Gene-Targeted Mice 

**DOI:** 10.1371/journal.pgen.0010023

**Published:** 2005-08-19

**Authors:** Ravi J Tolwani, Doug A Hamm, Liqun Tian, J. Daniel Sharer, Jerry Vockley, Piero Rinaldo, Dietrich Matern, Trenton R Schoeb, Philip A Wood

**Affiliations:** 1 Department of Genetics, University of Alabama, Birmingham, Alabama, United States of America; 2 Department of Comparative Medicine, Stanford University, Stanford, California, United States of America; 3 Department of Medical Genetics, Mayo Clinic College of Medicine, Rochester, Minnesota, United States of America; 4 Division of Medical Genetics, Children's Hospital, University of Pittsburgh, Pittsburgh, Pennsylvania, United States of America; 5 Laboratory Medicine and Pathology, Mayo Clinic College of Medicine, Rochester, Minnesota, United States of America; Johns Hopkins Institute, United States of America

## Abstract

Medium-chain acyl-CoA dehydrogenase (MCAD) deficiency is the most common inherited disorder of mitochondrial fatty acid β-oxidation in humans. To better understand the pathogenesis of this disease, we developed a mouse model for MCAD deficiency (MCAD^−/−^) by gene targeting in embryonic stem (ES) cells. The MCAD^−/−^ mice developed an organic aciduria and fatty liver, and showed profound cold intolerance at 4 °C with prior fasting. The sporadic cardiac lesions seen in MCAD^−/−^ mice have not been reported in human MCAD patients. There was significant neonatal mortality of MCAD^−/−^ pups demonstrating similarities to patterns of clinical episodes and mortality in MCAD-deficient patients. The MCAD-deficient mouse reproduced important aspects of human MCAD deficiency and is a valuable model for further analysis of the roles of fatty acid oxidation and pathogenesis of human diseases involving fatty acid oxidation.

## Synopsis

Medium-chain acyl-CoA dehydrogenase (MCAD) deficiency is one of the most common inherited disorders of metabolism. This defect in fatty acid oxidation can lead to severe and sometimes fatal disease, especially in young children because they are unable to tolerate a fasting episode. Metabolic complications include very low blood glucose concentrations and generation of toxic by-products. This disorder can result in sudden infant death. Using a process known as gene targeting in mouse embryonic stem cells, the authors have developed a mouse model with the same enzyme deficiency. This mouse model of MCAD deficiency develops many of the same disease characteristics found in affected children. The MCAD-deficient mouse model shows a high rate of newborn loss, intolerance to cold, and the characteristic biochemical changes in the blood, tissues, and urine that are very similar to those found in the human disease counterpart. The MCAD-deficient mouse model will allow researchers to better understand disease mechanisms so that new preventive measures or therapies can be developed.

## Introduction

Mitochondrial β-oxidation of fatty acids provides energy, especially during fasting conditions. Fatty acid oxidation occurs in mitochondria and consists of a repeating circuit of four sequential steps. There are four straight-chain acyl-CoA dehydrogenases involved in the initial step. Medium-chain acyl-CoA dehydrogenase (MCAD) (the mouse gene is *Acadm,* whereas the protein is MCAD), specifically, is responsible for catalyzing the dehydrogenation of medium-chain length (C_6_–C_12_) fatty acid thioesters [1]. *Acadm* is transcribed in the nucleus, translated in the cytosol, and translocated into the mitochondrial matrix [2–4]. Once inside the mitochondrial matrix, the MCAD monomers are assembled into homotetramers to gain enzymatic activity [4].

MCAD activity is essential for complete fatty acid oxidation. Inherited MCAD deficiency exists in humans as an autosomal recessive disorder. MCAD deficiency was first described in 1982—1983 [5–7] and has been described in numerous patients [1,8–11]. The carrier frequency in the Caucasian population has been estimated to be between 1 in 50 to 80 with an incidence of clinical disease expected at around 1 in 15,000 [1,9,12].

MCAD-deficient patients exhibit clinical episodes often associated with fasting. Patients manifest disease usually during the first two years of life. Symptoms include hypoketotic hypoglycemia and Reye-like episodes [1]. It is estimated that approximately 59% of patients presenting clinically between 15 to 26 mo of age die during their first clinical episode [1].

The pathogenesis of the wide range of metabolic disturbances in MCAD deficiency is poorly understood and certain aspects of patient management are controversial. An animal model for MCAD deficiency is essential to better understand the pathogenesis of MCAD deficiency and to develop better management regimens for human patients. To gain further insight into the mechanisms of this disease, we developed a mouse model of MCAD deficiency by gene targeting in embryonic stem (ES) cells (for reviews [13,14]). The mutant mice had many relevant features characteristic of the disease found in human MCAD-deficient patients, along with some unexpected findings.

## Results

### Gene Targeting and Generation of MCAD-Deficient Mice

MCAD insertion vector (MCAD IV2) was designed to undergo gap repair of the 1.3-kb deleted region upon homologous recombination in 129P2 (129P2/OlaHsd) ES cells E14–1. Correct targeting of the MCAD locus resulted in a duplication of exons 8, 9, and 10 and integration of flanking plasmid and Neo sequences (Figure 1A). The insertion vector was designed to duplicate exon 8, 9, and 10 at the MCAD locus. Translation of the duplicated exon 8 region results in the formation of premature stop codons resulting in truncation of the MCAD monomer. Specifically, the first premature stop codon arises after translation of only seven amino acids from the duplicated exon 8. The resulting MCAD monomer is missing the C-terminal domain α-helixes that are responsible for making intersubunit contacts to generate the functional MCAD homotetramer.

**Figure 1:**
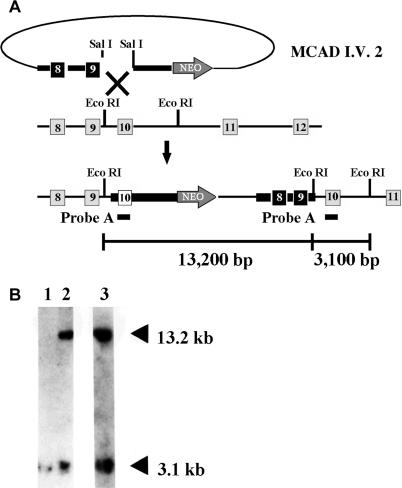
Strategy for Disruption of the Mouse *Acadm* Gene (A) The MCAD IV2 insertion targeting vector with a deleted 1.3-kb region encompassing exon 10 and flanking sequences. MCAD IV2 undergoes gap repair upon homologous recombination at the endogenous *Acadm* locus resulting in a duplication of exons 8, 9, and 10 at the disrupted allele. (B) Southern blot analysis of EcoRI-digested genomic DNA from ES cells screened by PCR. Probe A, a DNA fragment consisting of a portion of exon 10 that is not present in the targeting vector, hybridizes to an endogenous 3.1-kb fragment and, upon homologous recombination, to a 13.2-kb fragment. Lane 1 represents a wild-type ES cell line, and Lane 2 and 3 represent targeted ES cell lines.

ES cell clones were screened by PCR (data not shown) and confirmed by Southern blot analysis. Southern blot analysis used an exon 10 probe (probe A), not present in the targeting vector, hybridized to a 13.2-kb band in addition to the 3.1-kb endogenous band indicating targeted insertion of the vector at the *Acadm* locus (Figure 1B). Correctly targeted ES cell clones were microinjected into B6 (C57BL/6NTac) blastocysts to generate chimeric mice. Chimeric mice were backcrossed to both 129P2 and B6 inbred mice to produce MCAD^+/−^ and eventually MCAD^−/−^ mice on a B6/129 mixed background. The studies described here were conducted exclusively on the B6/129 mixed background compared with littermate controls or B6/129 control groups maintained by intercrosses as were the mutants. Perpetuating this mutation as a congenic mutant line on the 129P2 background proved impractical. The 129P2 mice were poor breeders as wild-types, and when introduced, the *Acadm* mutation was nearly lost on this background because of the high rate of neonatal death. Because of the molecular structure of the targeted allele, it proved virtually impossible to distinguish all three potential genotypes. We could clearly detect the presence or absence of the targeted allele, however, whether a particular mouse was MCAD^−/−^ or MCAD^+/−^ could not be determined by Southern blot or PCR of genomic DNA. Ultimately MCAD^−/−^ mice were ascertained by immunoblot analysis of offspring with subsequent perpetuation of MCAD^−/−^ and MCAD^+/+^ mice as separate groups.

### RNA Analysis

RT-PCR amplification from exon 7 to 11 from total heart RNA amplified the expected 600-base pair (bp) fragment in MCAD^+/+^ and MCAD^+/−^ mice, and a 1.5-kb fragment in MCAD^−/−^ mice (data not shown). Sequence analysis of the 1.5-kb fragment revealed that the amplified fragment consisted of exon 7 to exon 10 with 280 bp of pGEM plasmid sequence followed by exons 8–11. Some of the plasmid sequences, along with the pPGKNEOpA sequence, were deleted from this spliced mRNA.

Northern blot analysis revealed *Acadm* was normally expressed in all tissues analyzed from MCAD^+/+^ mice with the most robust expression occurring in brown fat, kidney, heart, skeletal muscle, and liver with minimal expression in the brain, white fat, and testes (Figure 2). Interestingly, although RT-PCR amplified an incorrectly spliced *Acadm* transcript, no *Acadm* transcripts were detected by northern blot analysis from MCAD^−/−^ mice. These results strongly suggest that the mutant RNA is unstable and degraded rapidly or, alternatively, undergoes nonsense mediated RNA decay.

**Figure 2:**
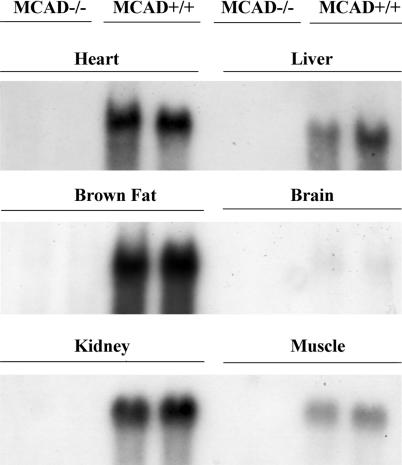
Northern Blot Analysis from MCAD^−/−^ (*n* = 2) and MCAD^+/+^ (*n* = 2) Mice *Acadm* message was detected from the heart, liver, brown fat, brain, kidney, and muscle (and white fat and testes, data not shown) of only MCAD^+/+^ mice. Most robust expression occurred in brown fat, kidney, heart, and skeletal muscle. MCAD^−/−^ mice had no detectable message in all tissues examined.

### Liver Enzyme Analyses

Immunoblot analyses of liver homogenates with anti-MCAD antisera demonstrated that the 42 kDa MCAD monomer was present in MCAD^+/+^ mice, but not in MCAD^−/−^ mice (Figure 3). As a control analysis, anti–short-chain acyl-CoA dehydrogenase (SCAD) antisera revealed no differences in levels of expression of SCAD protein between MCAD^+/+^ and MCAD^−/−^ mice (Figure 3).

**Figure 3:**
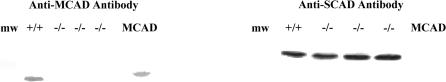
Immunoblots of Liver Homogenates from MCAD^+/+^ and MCAD^−/−^ Mice These were probed with anti-MCAD antibody or anti-SCAD antibody. Homozygous MCAD^−/−^ mice had no detectable MCAD protein. MCAD protein is only detectable under the MCAD protein–spiked (positive control) lane. As a control analysis, liver homogenates probed with anti-SCAD antibody revealed that SCAD protein was present in both MCAD^+/+^ and MCAD^−/−^ mice. No MCAD positive-control protein is detected by anti-SCAD antibodies (MCAD lane). mw, molecular weight standards.

Enzyme activity was analyzed in mouse liver homogenates using the electron transport flavoprotein (ETF) reduction assay with octanoyl-CoA (C_8:0_) and palmitoyl-CoA (C_16:0_) as substrates. MCAD^−/−^ mice had a significant reduction in ability to dehydrogenate octanoyl-CoA and a modest reduction in activity toward palmitoyl-CoA compared to MCAD^+/+^ mice (Table 1). Specifically, the dehydrogenation of octanoyl-CoA and palmitoyl-CoA substrates were reduced by 75% and by 30%, respectively, in MCAD^−/−^ mice as compared to MCAD^+/+^ controls.

**Table 1:**
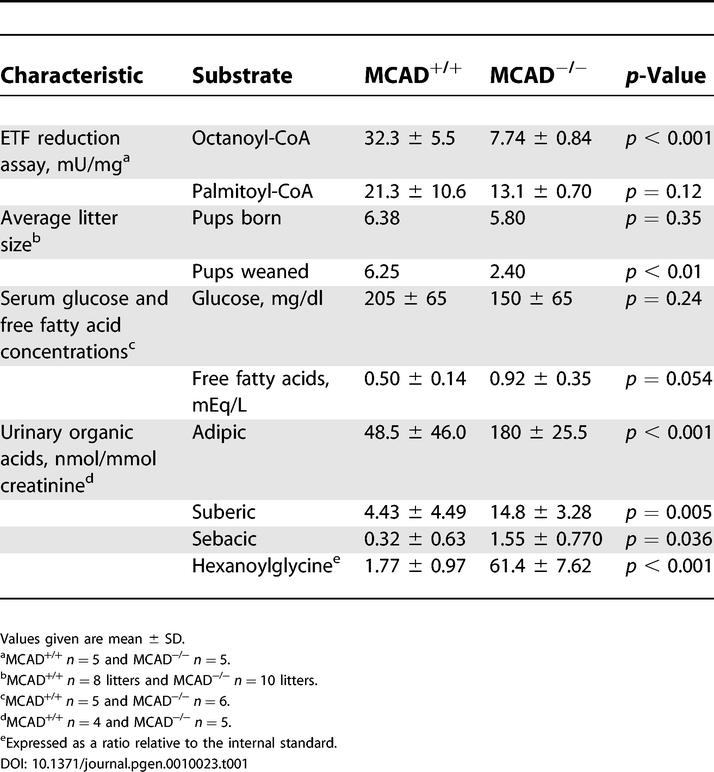
Characteristics of MCAD-Deficient Mice

Values given are mean ± SD.

^a^MCAD^+/+^ *n* = 5 and MCAD^−/−^ *n* = 5.

^b^MCAD^+/+^ *n* = 8 litters and MCAD^−/−^ *n* = 10 litters.

^c^MCAD^+/+^ *n* = 5 and MCAD^−/−^ *n* = 6.

^d^MCAD^+/+^ *n* = 4 and MCAD^−/−^ *n* = 5.

^e^Expressed as a ratio relative to the internal standard.

### Neonatal Deaths

Significant neonatal mortality was noted in MCAD^−/−^ pups. Although equal numbers of pups were born from MCAD^+/+^ and MCAD^−/−^ mice, only 41% of MCAD^−/−^ mice survived to weaning as compared to 98% of MCAD^+/+^ mice (Table 1). The mechanism of neonatal loss remains undetermined. The MCAD^−/−^ pups are abandoned more frequently than MCAD^+/+^ pups for unknown reasons. They are likely more prone to hypothermia than MCAD^+/+^. Because of the difficulty of distinguishing the MCAD^−/−^ mutants from the MCAD^+/−^ heterozygous pups by molecular analysis due to the insertion mutation, we could only compare MCAD^+/+^ × MCAD^+/+^ matings with MCAD^−/−^ × MCAD^−/−^ matings. Thus, we were unable to evaluate the pedigrees from heterozygous matings.

### Fasting and Cold Intolerance

In order to examine the stress effects of fasting on MCAD-deficient mice, they were fasted for 24 h prior to analysis. MCAD^−/−^ mice displayed lower serum glucose and elevated serum free fatty acid levels although neither result was significant, as compared to MCAD^+/+^ mice (Table 1).

To determine the effects of cold stress, mice were fasted for 18 h and placed in 4 °C environment for a 3-h period. The MCAD^−/−^ mice were significantly compromised within this short period of cold stress, some severe enough to result in fatalities. After 1 h of the cold challenge, the average rectal temperature of MCAD^−/−^ mice (*n* = 5) was 23.4 °C as compared with 35 °C for MCAD^+/+^ mice (*n* = 4). Rectal temperatures declined to unrecoverable temperatures of 16.7–19.2 °C in three of the five MCAD^−/−^ mice. By the end of the 1.5-h mark, the two surviving MCAD^−/−^ mice averaged 22.7 °C. In contrast, all four MCAD^+/+^ mice survived the 3-h cold stress, ending with an average rectal temperature of 33.6 °C.

### Organic Acid and Acylcarnitine Analysis

Urine organic acid analysis revealed that MCAD^−/−^ mice developed an organic acid profile similar to MCAD-deficient human patients. Specifically, when fasted for 18 h, MCAD^−/−^ mice developed significantly elevated concentrations of adipic, suberic, and sebacic acids and hexanoylglycine as compared to MCAD^+/+^ controls, which showed trace to no detectable amounts of the same organic acids (Table 1). Adipic acid is not specific to MCAD deficiency. We also evaluated β-hydroxybutyric and acetoacetic concentrations and found no significant differences between MCAD genotypes (data not shown).

Comparison of MCAD^+/+^ and MCAD^−/−^ mice revealed no significant differences in total serum carnitine concentrations between MCAD^+/+^ and MCAD^−/−^ mice, but MCAD^−/−^ mice had a 5- to 6-fold elevation of serum decenoylcarnitine evident in the acylcarnitine profile (Figure 4A). Bile acylcarnitine analysis revealed a similar acylcarnitine pattern as in serum (Figure 4B). However, the acylcarnitine profiles of the MCAD^−/−^ mice are different from those of human MCAD-deficient patients (Figure 4C). Human patients present with elevated levels of C_6_, C_8_, and C_10:1_ acylcarnitines, as did the mutant mice; however, the predominant peak was C_8_ acylcarnitine in humans, whereas in the mouse it was C_10:1_ acylcarnitine.

**Figure 4:**
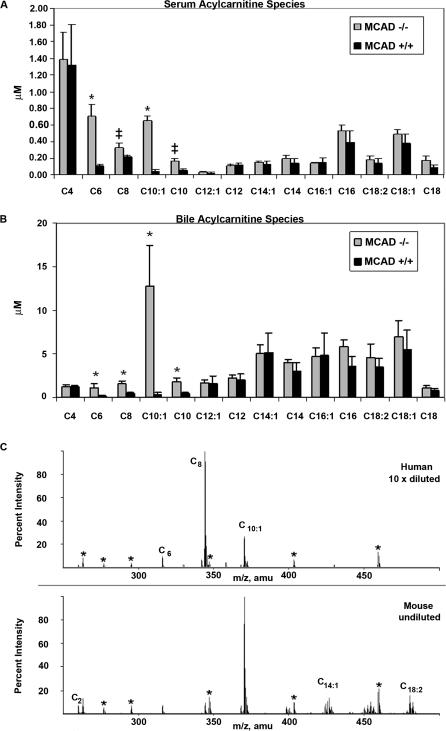
Acylcarnitine Analyses (A) Serum acylcarnitine analysis of MCAD^+/+^ (*n* = 4) and MCAD^−/−^ mice (*n* = 4) There are significant elevations in acylcarnitine species as indicated in MCAD^−/−^ mice. Values shown are mean values ± standard deviation (SD). Asterisk indicates *p* < 0.002 and ‡ indicates *p* < 0.01. (B) There are significant elevations in bile acylcarnitines of the same mice shown in (A) as indicated. Values shown are mean values ± SD. Asterisk indicates *p* < 0.001. (C) Bile acylcarnitine profile of an MCAD^−/−^ mouse compared to a human patient with MCAD deficiency. Internal standards are indicated by an asterisk.

### Histopathology

Complete histopathologic examination of one group of mutant and MCAD^+/+^ control mice after a 24-h fast demonstrated diffuse microvesicular and macrovesicular hepatic steatosis in 6–8-wk-old MCAD^−/−^ mice whereas MCAD^+/+^ mice had no histologic changes (Figure 5A and 5B). In another group of 4-wk-old MCAD^+/+^ and MCAD^−/−^ mice fasted for 24-h, there were minimal to no abnormalities in all organs evaluated. Only the older MCAD^−/−^ mice, therefore, demonstrated hepatic steatosis. In addition, we found sporadic cardiac lesions in multiple MCAD^−/−^ mice.

**Figure 5:**
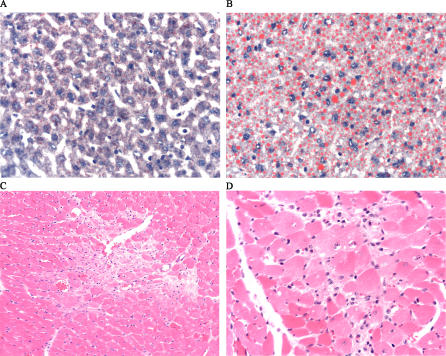
Histopathology of MCAD^+/+^ and MCAD^−/−^ Mice (A) MCAD^+/+^ mice had no evidence of hepatic steatosis following a 24-h fast. Liver section with Oil-Red O stain. (B) Hepatosteatosis in MCAD^−/−^ mouse following a 24-h fast. Oil-Red O stained liver sections revealed severe and diffuse microvesicular and macrovesicular hepatic steatosis in MCAD^−/−^ mice. (C and D) Diffuse cardiomyopathy with chronic active multifocal myocyte degeneration and necrosis in MCAD^−/−^ mice.

In one example, an MCAD^−/−^ mouse had diffuse cardiomyopathy with chronic active multifocal myocyte degeneration and necrosis (Figure 5C and 5D). Changes in degenerating myocytes included swelling, pale staining, and, in portions of the sarcoplasm, replacement of myofibrils with finely granular eosinophilic material. Nuclei of affected myocytes were large, pale, and vesicular and had prominent nucleoli. In the most severely affected areas, there was loss of myocytes accompanied by fibrosis. In the wall of the aorta at the base of the heart there was multifocal degeneration of the elastic tissue, accompanied by multifocal collections of globular translucent yellow-brown pigment interpreted to be ceroid/lipofuscin. Similar deposits were scattered within adjacent adipose tissue.

## Discussion

Successfully targeting *Acadm* produced a mouse model for MCAD deficiency with features that mimic the clinical, biochemical, and pathologic phenotype found in human patients. MCAD-deficient patients have abnormal plasma and urine metabolites associated with the medium chain–length enzyme specificity. MCAD-deficient patients [15] often display a characteristic urinary hexanoylglycine peak, as was seen in MCAD^−/−^ mice. Acylcarnitine analysis indicated, however, mouse MCAD is more active toward longer chain substrates than the human MCAD enzyme. This finding is similar to that seen with very long-chain acyl-CoA dehydrogenase (VLCAD) where mouse VLCAD is most active toward C_16_ acyl-substrates as compared to human VLCAD with the most enzymatic activity toward C_14_ acyl-substrates [16].

ETF reduction assays of liver homogenates were performed to characterize the MCAD^−/−^ mice at the enzymatic level. MCAD^−/−^ mice had a significantly reduced ability to dehydrogenate C_8_-CoA, as is the case in human patients where MCAD activity is reduced to near zero with C_8_-CoA . This was less so with palmitoyl-CoA (C_16:0_). Because there was clearly no MCAD antigen detected in MCAD^−/−^ mice, the residual dehydrogenase activity measured with these two substrates must represent the activity of other chain length–specific acyl-CoA dehydrogenases.

A high degree of neonatal mortality in MCAD^−/−^ mice was a striking observation and appears to be analogous to the patterns of clinical episodes and mortality in MCAD-deficient patients. MCAD^−/−^ mice exhibited significant neonatal mortality with approximately 60% of the MCAD^−/−^ pups dying prior to weaning at 3 wk of age. Human patients present with hypoglycemia, hypoketonemia, and nonketotic organic aciduria precipitated by fasting, most frequently during the first 24 mo in life [1]. It is likely that neonatal MCAD^−/−^ pups are manifesting sensitivity to fasting with decompensation in a short period of time if maternal milk is not ingested. In contrast, no mortality was noted in adult MCAD^−/−^ mice unless challenged with cold stress and fasting. Under cold challenge conditions, however, MCAD^−/−^ mice were unable to maintain body temperature. Brown fat is predominantly responsible for thermogenesis and normally expresses high levels of *Acadm* mRNA.

The microvesicular and macrovesicular hepatic steatosis seen in fasted MCAD^−/−^ mice is consistent with the primary pathological finding seen in human MCAD patients with fasting stress. Sporadic cardiac lesions in MCAD^−/−^ mice, however, were an interesting and unexpected finding. The diffuse cardiomyopathy with multifocal myocyte degeneration and necrosis observed in MCAD^−/−^ mice has not been reported in human MCAD patients, however, cardiac arrhythmias and dysfunction have been reported in MCAD-deficient patients [17, 18]. Interestingly, cardiomyopathy has been observed in VLCAD deficiency [19] and other disorders of long chain fat metabolism such as severe CPT-1 and -2 deficiencies [1]. Thus it is tempting to relate the cardiac problems in the mouse to the apparent broader range of substrate utilization of mouse MCAD. The inconsistent liver and cardiac lesions in these mice is analogous with the significant inter- and intrafamilial phenotypic heterogeneity seen in MCAD deficiency in humans [1,20].

In comparisons with our experiences with the other mouse models for acyl-CoA dehydrogenase deficiencies, the overall phenotype of MCAD^−/−^ mice is less severe than that found in LCAD^−/−^ mice, yet more pronounced than the VLCAD^−/−^ or SCAD^−/−^ mouse models [16,21,22]. All of these mutants are cold intolerant and display varying degrees of fatty changes in liver, heart, and kidney. LCAD^−/−^ mice show more spontaneous deaths and gestational losses than the other deficiencies [21]. The significant neonatal mortality in MCAD^−/−^ mice is distinctive from these other mouse models suggesting a greater degree of sensitivity to fasting intolerance. The phenotypes of both the VLCAD^−/−^ and SCAD^−/−^ mice are relatively mild if the animals are not cold stressed [16,22]. The MCAD-deficient mouse offers new insights into the pathogenesis of mitochondrial β-oxidation deficiencies and will provide a robust tool to better understand the role of fatty acids in other relevant diseases.

## Materials and Methods

### Construction of MCAD targeting vector.

A neomycin resistance gene cassette [23] was subcloned into the SalI site of pGEM11Zf(+). The plasmid was digested with EcoRI and the overhangs were filled with Klenow enzyme. Subsequent ligation of the blunt ends recircularized the vector and destroyed the EcoRI site within the polylinker of the pGEMl1Zf(+) plasmid. Next, an 8-kb *Acadm* genomic fragment spanning exons 8, 9, and 10 and flanking intron sequences, originally obtained from a Lambda FIXII 129Sv mouse genomic library [24,25], was directionally cloned into the NotI and XhoI sites of pGEM11Zf(+). The vector was digested with BamHI and EcoRI to remove a 1.3-kb BamHI/EcoRI genomic fragment containing exon 10 and flanking intron sequences. The digested vector, without the 1.3-kb BamHI/EcoRI genomic fragment, was purified by gel purification and recircularized by ligation using three oligonucleotides: 5′-AATTGTCGACA-3′; 5′GATCGTCGACA-3′; and 5′-TCGATGTCGAC-3′. The recircularized vector, resulting from the ligation of the long arm to the short arm of homology, contained a unique SalI site where the 1.3-kb exon 10 region was deleted.

### Generation of MCAD-deficient mice.

The *Acadm* insertion vector was linearized by SalI digestion, the site of the 1.3-kb genomic fragment deletion, and electroporated into E14–1 ES cells (a kind gift from R. Kuhn), derived from 129P2 mice. Correctly targeted *Acadm* insertion vector was designed to undergo gap repair of the 1.3-kb deletion upon double stranded–break repair [26] during homologous recombination. Southern blot analysis was conducted to confirm homologous recombination. Genomic DNA from G418 resistant ES colonies was digested with EcoRI, electrophoresed, blotted, and probed with an 850-bp probe (probe A) generated by PCR from *Acadm* exon 10 to intron 10. This DNA fragment is not present within *Acadm* insertion vector and was expected to hybridize to a 13.2-kb genomic DNA fragment upon homologous recombination. Correctly targeted ES cell clones were microinjected into B6 blastocysts to generate chimeric mice. Chimeric mice were subsequently backcrossed to B6 and 129P2 mice to produce gene-targeted mice *Acadm^tmUab1/+^* (MCAD^+/−^) and eventually *Acadm^tmUab1/tm1Uab^* MCAD^−/−^ (B6;129) mice. Mice were negative for mouse pathogens based on serological assays for ten different viruses, aerobic bacterial cultures of nasopharynx and cecum, examinations for endo- and ectoparasites, and histopathology of all major organs.

### RNA analysis.

Total RNA was isolated from the heart of 30 day old MCAD^+/+^, MCAD^+/−^, and MCAD^−/−^ mice by standard techniques using guanidinium thiocyanate method [27]. Reverse transcription was performed using random oligonucleotides as recommended by the manufacturer (Clontech, Mountain View, California, United States). PCR was subsequently performed using oligonucleotides specific to exon 7 and exon 11 of *Acadm*. PCR amplifications were performed as described above. PCR fragments were subsequently sequenced after subcloning into pGEM-T Easy vector (Promega, Madison, Wisconsin, United States).

In order to determine the extent of *Acadm* mRNA expressed from the MCAD^−/−^ mice, northern blot analysis was performed. Total RNA was isolated from heart, liver, brown fat, brain, kidney, skeletal muscle, white fat, and testes of 3-mo-old MCAD^+/+^ and MCAD^−/−^ mice using the Ultraspec RNA Isolation Kit (BIOTEX Laboratories, Inc., Houston, Texas, United States) as per manufacturer's protocol. Ten μg of total RNA from each sample was loaded onto a 0.8% agarose-formaldehyde gel, transferred to nitrocellulose filter (Hybond N; GE Healthcare Amersham Biosciences Corp., Piscataway, New Jersey, United States), and hybridized with ^32^P-radiolabeled full-length mouse *Acadm* cDNA probe using standard procedures [28]. Hybridizations were performed under highly stringent conditions (42 °C in 2× SSC, 50% formamide, 10% dextran sulfate, 5× Denhardt's reagent, 1% SDS, and salmon sperm DNA) for 18 h. The hybridized filter was washed two times in 4× SSC, 0.1% SDS and two times in 2× SSC; 0.1% SDS at 55 °C for 1 h. The filter was exposed to autoradiographic film (Hyperfilm MP; GE Healthcare Amersham Biosciences, Piscataway, New Jersey, United States). Replicate agarose-formaldehyde gels were stained by ethidium bromide to verify equal RNA loading.

### Immunoblot analysis of MCAD protein.

To evaluate the quantity of MCAD protein in mouse tissue, liver samples from 6–8-wk-old MCAD^+/+^ (*n* = 1) and MCAD^−/−^ (*n* = 3) mice were immediately frozen in liquid nitrogen and stored at −80 °C. For analysis, tissue was homogenized and lysed in RIPA buffer (1× PBS, 1% Nonidet P-40, 0.5% sodium deoxycholate, and 1% SDS) with 10% glycerol and Complete Protease Inhibitor, (Roche Diagnostics Corporation, Indianapolis, Indiana, United States), 1 mM of phenylmethylsulfonylfluoride, and 1 mM of sodium orthovanadate. Lysates were quantified by Bradford BCA protein assay (Bio-Rad, Hercules, California, United States). Protein lysates were denatured, separated in 8% SDS-PAGE, and transferred overnight onto a 0.45 μm nitrocellulose membrane (Schleicher and Schuell, Keene, New Hampshire, United States). After blocking with 5% nonfat milk in phosphate-buffered saline with 0.1% Tween-20, the membrane was immunoblotted overnight at 4 °C with 1:500 dilution of an anti-MCAD antibody. Blots were incubated in anti-mouse IgG HRP-conjugated secondary antibody for 2–4 h at room temperature using standard procedures and developed by chemiluminiscence (Renaissance, NEN Lifesciences Products, Boston, Massachusetts, United States).

### Liver enzyme activity.

In order to evaluate MCAD activity in mice, liver homogenates were prepared from MCAD^+/+^ (*n* = 5) and MCAD^−/−^ mice (*n* = 5). The sensitive and highly specific anaerobic ETF reduction assay was used on tissue extracts with octanoyl-CoA (C_8_) and palmitoyl-CoA (C_16_) as substrate as previously described [29].

### Fasting and cold challenge.

Eight-wk-old MCAD^+/+^ and MCAD^−/−^ mice were fasted for 18 h (cold tolerance experiments) or 24 h (serum glucose, free fatty acid, organic acid, and carnitine experiments) prior to analysis. Glucose concentration was measured in 10 μl sera using an Ektachem DT II system (Johnson and Johnson Clinical Diagnostics, Rochester, New York, United States). Total non-esterified fatty acids (NEFA) were measured by an enzymatic, colorimetric method (“NEFA-C” reagents, Wako Diagnostics, Richmond, Virginia, United States). The assay was modified to accommodate a reduced sample size (10 μl) and use of a microplate reader for measurement of optical density at 550 nm. Urine organic acid analyses were performed using gas chromatography-mass spectroscopy as previously described [22,30], except tetracosane (C_24_; Sigma, St. Louis, Missouri, United States) was used as the internal standard, and quantitative determinations were made based on comparison with synthetic calibration standards. Acylcarnitine analyses in serum and bile were conducted using electrospray tandem mass spectrometry [31,32].

### Histopathology.

Twelve mice were examined for gross and histologic abnormalities, including one male and one female MCAD^−/−^ mouse 18-mo-old, one male and one female MCAD^+/+^ mouse 6-mo-old, two male and two female MCAD^−/−^ mice 4-wk-old, and two male and two female MCAD^+/+^ mice 4-wk-old. Kidney, spleen, pancreas, liver, brain, heart, testicles, ovaries, and skeletal muscle were fixed by immersion in buffered 10% formalin, processed routinely for paraffin sectioning, sectioned at 5 μm, and stained with hematoxylin and eosin. Frozen liver sections were prepared using standard methods and sections were stained with Oil-red-O. Slides were examined without knowledge of genotype or age. Other mice were examined because of sporadic clinical disease. Those with visible cardiac enlargement were evaluated for cardiac lesions.

### Statistical analyses.

Results between groups were tested for statistical significance using Student's *t*-test. A *p* < 0.05 was set as significant.

## Supporting Information

### Accession Number

The GenBank (http://www.ncbi.nlm.nih.gov/Genbank) accession number for the mouse gene *Acadm* is U07159.

## Abbreviations

bp: base pair
ES: embryonic stem
ETF: electron transport flavoprotein
MCAD: medium-chain acyl-CoA dehydrogenase
SCAD: short-chain acyl-CoA dehydrogenase
SD: standard deviation
VLCAD: very long-chain acyl-CoA dehydrogenase

## References

[pgen-0010023-b01] Rinaldo P, Matern D, Bennett MJ (2002). Fatty acid oxidation disorders. Annu Rev Physiol.

[pgen-0010023-b02] Matsubara Y, Kraus JP, Ozasa H, Glassberg R, Finocchiaro G (1987). Molecular cloning and nucleotide sequence of cDNA encoding the entire precursor of rat liver medium chain acyl coenzyme A dehydrogenase. J Biol Chem.

[pgen-0010023-b03] Kelly DP, Kim JJ, Billadello JJ, Hainline BE, Chu TW (1987). Nucleotide sequence of medium-chain acyl-CoA dehydrogenase mRNA and its expression in enzyme-deficient human tissue. Proc Natl Acad Sci U S A.

[pgen-0010023-b04] Ikeda Y, Keese SM, Fenton WA, Tanaka K (1987). Biosynthesis of four rat liver mitochondrial acyl-CoA dehydrogenases: In vitro synthesis, import into mitochondria, and processing of their precursors in a cell-free system and in cultured cells. Arch Biochem Biophys.

[pgen-0010023-b05] Kolvraa S, Gregersen N, Christensen E, Hobolth N (1982). In vitro fibroblast studies in a patient with C6-C10-dicarboxylic aciduria: Evidence for a defect in general acyl-CoA dehydrogenase. Clin Chim Acta.

[pgen-0010023-b06] Stanley CA, Hale DE, Coates PM, Hall CL, Corkey BE (1983). Medium-chain acyl-CoA dehydrogenase deficiency in children with non-ketotic hypoglycemia and low carnitine levels. Ped Res.

[pgen-0010023-b07] Rhead WJ, Amendt BA, Fritchman KS, Felts SJ Dicarboxylic aciduria: Deficient [1-^14^C] octonoate oxidation and medium-chain acyl-CoA dehydrogenase in fibroblasts. Science.

[pgen-0010023-b08] Coates PM (1992). Historical perspective of medium-chain acyl-CoA dehydrogenase deficiency: A decade of discovery. Prog Clin Biol Res.

[pgen-0010023-b09] Matsubara Y, Narisawa K, Tada K (1992). Medium-chain acyl-CoA dehydrogenase deficiency: Molecular aspects. Eur J Pediatr.

[pgen-0010023-b10] Ding JH, Bross P, Yang BZ, Lafolla AK, Millington DS, Coates PM, Tanaka K (1992). Genetic heterogeneity in MCAD deficiency: Frequency of K329E allele and identification of three additional mutant alleles. New developments in fatty acid oxidation.

[pgen-0010023-b11] Coates PM, Tanaka K, Workshop on Molecular Aspects of MCAD Deficiency (1992). Mutations causing medium-chain acyl-CoA dehydrogenase deficiency: A collaborative compilation of the data from 172 patients. New developments in fatty acid oxidation.

[pgen-0010023-b12] Matern D Rinaldo P 2003 Medium-chain acyl-coenzyme A dehydrogenase deficiency Available: http://www.geneclinics.org/servlet/access?db=geneclinics&site=gt&id=8888891&key=YpeEakmrXPZ3i&gry=&fcn=y&fw=nYYD&filename=/profiles/mcad/index.html. Accessed 21 July 2005.

[pgen-0010023-b13] Capecchi MR (1989). Altering the genome by homologous recombination. Science.

[pgen-0010023-b14] Koller BH, Smithies O (1992). Altering genes in animals by gene targeting. Annu Rev Immunol.

[pgen-0010023-b15] Rinaldo P, O'Shea JJ, Coates PM, Hale DE, Stanley CA (1988). Medium-chain acyl-CoA dehydrogenase deficiency. Diagnosis by stable-isotope dilution measurement of urinary n-hexanoylglycine and 3-phenylpropionylglycine. N Engl J Med.

[pgen-0010023-b16] Cox KB, Hamm DA, Millington DS, Matern D, Vockley J (2001). Gestational, pathologic and biochemical differences between very long-chain acyl-CoA dehydrogenase deficiency and long-chain acyl-CoA dehydrogenase deficiency in the mouse. Hum Mol Genet.

[pgen-0010023-b17] Feillet F, Steinmann G, Vianey-Saban C, de Chillou C, Sadoul N (2003). Adult presentation of MCAD deficiency revealed by coma and severe arrythmias. Intensive Care Med.

[pgen-0010023-b18] Maclean K, Rasiah VS, Kirk EP, Carpenter K, Cooper S (2005). Pulmonary haemorrhage and cardiac dysfunction in a neonate with medium-chain acyl-CoA dehydrogenase (MCAD) deficiency. Acta Paediatr.

[pgen-0010023-b19] Vianey-Saban C, Divry P, Brivet M, Nada M, Zabot MT (1998). Mitochondrial very-long-chain acyl-coenzyme A dehydrogenase deficiency: Clinical characteristics and diagnostic considerations in 30 patients. Clin Chim Acta.

[pgen-0010023-b20] Duran M, Hofkamp M, Rhead WJ, Saudubray JM, Wadman SK (1986). Sudden child death and ‘healthy' affected family members with medium-chain acyl-coenzyme A dehydrogenase deficiency. Pediatrics.

[pgen-0010023-b21] Kurtz DM, Rinaldo P, Rhead WJ, Tian L, Millington DS (1998). Targeted disruption of mouse long-chain acyl-CoA dehydrogenase gene reveals crucial roles for fatty acid oxidation. Proc Natl Acad Sci U S A.

[pgen-0010023-b22] Wood PA, Amendt BA, Rhead WJ, Millington DS, Inoue F (1989). Short-chain acyl-coenzyme A dehydrogenase deficiency in mice. Pediatr Res.

[pgen-0010023-b23] Soriano P, Montgomery C, Geske R, Bradley A (1991). Targeted disruption of the c-src proto-oncogene leads to osteopetrosis in mice. Cell.

[pgen-0010023-b24] Tolwani RJ, Farmer SC, Wood PA (1994). Molecular cloning and characterization of the mouse medium-chain acyl-CoA dehydrogenase cDNA. Genomics.

[pgen-0010023-b25] Tolwani RJ, Farmer SC, Johnson KR, Davisson MT, Kurtz DM (1996). Structure and chromosomal location of the mouse medium-chain acyl-CoA dehydrogenase-encoding gene and its promoter. Gene.

[pgen-0010023-b26] Szostak JW, Orr-Weaver TL, Rothstein RJ, Stahl FW (1983). The double-strand-break repair model for recombination. Cell.

[pgen-0010023-b27] Chomczynski P, Sacchi N (1987). Single-step method of RNA isolation by acid guanidinium thiocyanate-phenol-chloroform extraction. Anal Biochem.

[pgen-0010023-b28] Sambrook J, Fritsch EF, Maniatis T (1989). Molecular cloning: A laboratory manual, 2nd ed.

[pgen-0010023-b29] Mohsen AW, Vockley J (1995). Identification of the active site catalytic residue in human isovaleryl-CoA dehydrogenase. Biochemistry.

[pgen-0010023-b30] Wood PA, Armstrong D, Sauls D, Davisson MT (1988). Screening mutant mice for inborn errors of metabolism. Lab Anim Sci.

[pgen-0010023-b31] Rashed MS, Ozand PT, Bucknall MP, Little D (1995). Diagnosis of inborn errors of metabolism from blood spots by acylcarnitines and amino acids profiling using automated electrospray tandem mass spectrometry. Pediatr Res.

[pgen-0010023-b32] Rinaldo P, Hahn SH, Matern D, Burtis CA, Ashwood ER, Bruns D (2005). Inborn errors of amino acid, organic acid, and fatty acid metabolism. Tietz textbook of clinical chemistry, 4th ed..

